# Perceptions of how decent work affects employee retention in a South African financial services institution

**DOI:** 10.3389/fsoc.2026.1744379

**Published:** 2026-03-11

**Authors:** Patricia Lungile Vilakazi, Wilfred Isioma Ukpere, Musawenkosi Donia Saurombe

**Affiliations:** Department of Industrial Psychology and People Management, College of Business and Economics, University of Johannesburg, Johannesburg, South Africa

**Keywords:** decent work, employee retention, financial services sector, precarious work, temporary employment

## Abstract

**Introduction:**

The financial services sector in South Africa faces unprecedented challenges regarding employee retention, with high turnover rates threatening organisational sustainability and competitive advantage. Increasingly flexible employment has resulted in more casual labor, often characterized by deficient decent work practices like poor remuneration, which disfavor successful employee retention. This research aimed to understand perceptions of how decent work affects employee retention in a South African financial services institution. The ILO encapsulates decent work as productive employment characterized by equity, freedom, human dignity and security. Understanding how decent work perceptions affect retention is essential for organisations seeking to develop evidence-based strategies for effective talent management and sustainable organisational development in an increasingly challenging labor market.

**Methods:**

A qualitative approach and case study strategy allowed us to explore participants’ lived experiences regarding how decent work affects retention in the selected South African financial services institution. Purposive sampling was employed and semi-structured interviews were conducted with 17 employees from various departments within the organisation explored. Thematic analysis was used to analyse the data.

**Results:**

Four perceptions of decent work were developed including security-based perceptions, emphasizing employment stability; relationship-centred perceptions, focusing on supportive management and workplace relationships; compensation and recognition-focused perceptions, highlighting fair remuneration and acknowledgement; and development and growth-oriented perceptions, emphasizing professional advancement opportunities. Six critical retention factors were identified including career development and growth opportunities, recognition and reward systems, work-life balance and flexible work arrangements, compensation and benefits structure, job security and employment stability, as well as alignment of company culture and values.

**Discussion:**

This research provides evidence-based insights for developing comprehensive retention strategies within the South African financial services institution by promoting decent work practices. Key organisational recommendations include establishing clear pathways from temporary to permanent employment, implementing inclusive development programmes accessible to all employees, creating transparent compensation structures with comprehensive benefits, institutionalising flexible work arrangements, and developing multi-layered recognition systems.

## 1 Introduction

The financial services sector plays a crucial role in South Africa’s economy, boasting an asset-to-GDP ratio that is significantly higher than that of other emerging nations (Financial Services Compliance Authority, 2022). The sector encompasses banking, insurance, and pension funds, with the majority of assets held by the banking industry. South Africa has been praised for having a well-developed and resilient financial services sector amid recent global financial market volatility (IMF Executive Board Consultations, 2023). However, beneath this apparent stability lies a growing crisis in human capital management that threatens the sector’s long-term sustainability.

Talent attraction and retention have emerged as critical challenges facing the financial services industry globally and South Africa is no exception (Ngobeni et al., 2022; Sija, 2021). The goal of employee retention extends beyond mere numbers, as organisations seek to retain top talent as an invaluable asset that supports organisational performance while promoting a competitive advantage (Sepahvand and Khodashahri, 2021). Van der Merwe et al. (2020) report alarming turnover intentions among employees in South African financial institutions, emphasising the urgent need for targeted retention interventions. The Crowe Bank Compensation and Benefits Survey (2023) reveals that banks continue to struggle with retaining younger talent, with poor compensation, limited benefits, and a lack of career advancement identified as primary drivers of turnover. Meanwhile, Villacé-Molinero et al. (2024) found that women often leave due to limited work-life balance, thus necessitating more customized retention strategies that cater to the needs of employees from different backgrounds and walks of life.

It is worth noting that employees who perceive their work environment as conducive demonstrate higher job satisfaction, better well-being, and greater engagement (Duffy et al., 2021; Işık et al., 2019). Job dissatisfaction and unfavourable working conditions remain primary drivers of attrition (Maloba and Pillay-Naidoo, 2022). Chada et al.'s (2022) study found a favourable association between decent work and employee engagement, suggesting that organisations should offer jobs that promote work-life balance, safety, health, and adequate pay. Conversely, undesirable work conditions contribute significantly to turnover intentions (Wang et al., 2019a).

Meanwhile, the financial implications of employee turnover extend beyond the immediate recruitment costs. Hom et al. (2019) identify multiple consequences, including high recruitment fees, training and development costs for new staff, and lost income. Recruitment expenses alone account for 50–60% of first-year compensation (Sepahvand and Khodashahri, 2021). Additionally, reduced productivity results from staff losses as surviving members become overworked (Hughes et al., 2021). These factors underscore the critical importance of understanding and addressing retention challenges through the principles of decent work.

Recent revenue decline, technological advancements, and the gradual transition from traditional employment models to precarious work arrangements presents significant challenges for both employees and organisations in the financial services sector (Esieboma, 2020). While this shift offers flexibility for gig and seasonal employment seekers, it creates substantial obstacles for workers in terms of reduced employee productivity and employment stability, as well as deteriorating employee benefits, and reduced professional advancement opportunities (Blustein et al., 2019; Demiral et al., 2022). Dabai and Novel’s (2022) study reveals that the banking sector is increasingly replacing permanent staff with temporary labour to reduce costs, subjecting casual workers to poor or non-existent social benefits, high job insecurity, short tenure, low pay, and significant health and safety risks. According to the Government of Western Australia (2025), casual work is typically characterized by irregular work with no guarantee of long-term continuation; thus, such work is considered precarious. Meanwhile, precarious employment fundamentally undermines the principles of Sustainable Development Goal 8, which advocates for decent work and economic growth (Hughes et al., 2021; Mpofu, 2023).

The International Labour Organisation (ILO) established the Decent Work Framework to address deteriorating employment conditions, promoting work that encompasses acceptable standards while protecting vulnerable employees from exploitation (ILO, 1999). This framework, which is integrated into the 2030 Sustainable Development Goals agenda, outlines principles for promoting fair labour practices and protecting human rights in the workplace. Organisations failing to comply with fair labour practices risk governmental sanctions (UN, 2015). The decent work framework’s four fundamental values, namely freedom, equity, security, and human dignity, provide a foundation for understanding employment quality in contemporary organisations.

While numerous global studies specifically highlight the retention challenges associated with deficient decent work practices (Chada et al., 2022; Chikove and Hofisi, 2025; Kaan Namal et al., 2024; Wang et al., 2019a; Zehri et al., 2024), the link between these two constructs is mostly merely implied in research within South African studies, especially regarding the financial services sector. For instance, while decent work-related aspects such as fair remuneration and conducive organisational culture emerge as crucial to enhancing employee retention, these aspects are not explicitly framed according to the ILO’s definition of decent work (Maloba and Pillay-Naidoo, 2022; Maphanga and Olivier, 2025; Potgieter and Snyman, 2018; van der Merwe et al., 2020), thus, the contribution of these studies to the research topic is inadvertent. Saurombe and Barkhuizen (2022) also investigated the link between happiness, meaningfulness (a key element of decent work encapsulated by the ILO) and the intention to quit; however, decent work is not explicitly implicated in their research. Hammond and Coetzee (2022) further emphasised that retention enablers include conducive work conditions, fair compensation, and continuous career development opportunities; however, the study does not directly link these retention enablers to decent work. Another shortcoming we identified in extant literature is that research on how decent work affects retention tends to be concentrated in other sectors such as hospitality, where precarious work has been more rampant over the years (Deery and Jago, 2015; Gupta, 2019; Wang and Cheung, 2024), while sectors like the financial services remain understudied so far. Hence, this research sought to contribute toward addressing these gaps, amid recently increasing interest in decent work-related studies (especially in the last 5–10 years), though such studies remain inadequate in specifically addressing decent work in the South African financial services sector (Blustein et al., 2019; van der Merwe et al., 2020).

### 1.1 Research problem

Globalisation and the Fourth Industrial Revolution (4IR) have changed South Africa’s employment landscape. Since the dawn of globalisation and the recent 4IR, there seems to be continuous striving in respect of labour standards, which affects decent work and workers’ working conditions negatively, particularly in the financial services sector. In recent times, decent work and job security, particularly in the financial services sector, seems to have been replaced with precarious jobs such as casual jobs, temporary jobs, contract jobs, and labour brokering (Dabai and Novel, 2022). In fact, current 4IR rapid technological advancement seems to have accelerated technological displacement of workers through automation, downsizing, redundancy, and retrenchment, all of which are antithetical to decent work and could affect employees’ retention negatively in South Africa’s financial services sector (Nesindande et al., 2025; Ngobeni et al., 2022).

### 1.2 Research purpose and objectives

This research aimed to explore the multifaceted nature of decent work as experienced by employees in the South African context and how these perceptions influence such employees’ decisions to stay with their organisation. The research focused on a single financial services institution in South Africa, wherein retention was observed to dwindle over time, due to deficient decent work-related factors.

The specific objectives were to understand the perceptions of how decent work affects employee retention in a South African financial services institution, and to identify factors that contribute to the retention of employees in the financial services institution.

## 2 A brief literature review

### 2.1 Theoretical foundation

This research is theoretically underpinned by the decent work framework which elucidates the conceptualization of decent work and its adoption in this study, focusing on key constructs such as fair compensation, job security and career advancement, while the social exchange theory is used to explain how employees’ retention tends to increase when they feel their employers provide them with a decent working experience.

#### 2.1.1 The decent work framework

The International Labour Organisation’s decent work framework provides the theoretical foundation for understanding employment quality in contemporary organisations. Established in 1999, this framework addresses the detrimental effects of working conditions while promoting employment that encompasses acceptable standards. The framework’s integration into the 2030 Sustainable Development Goals agenda, specifically SDG 8, underscores its global significance in promoting fair labour practices and protecting human rights in the workplace (ILO, 1999; UN, 2015).

The decent work framework is guided by four fundamental values: freedom, equity, security, and human dignity (ILO, 1999). These values support strategic objectives that advocate for working conditions that respect employees’ fundamental rights, while creating safe work environments, enabling freedom of speech, and fostering collaboration to improve policies and procedures (ILO, 2022). The framework’s primary objective is to extend social protection coverage to all workers, including those in informal sectors, by formulating rules and implementing programs that establish protective measures for workers and families at risk (Rantanen et al., 2020).

Decent work encompasses multiple dimensions, including preserving people’s rights, enhancing working conditions, ensuring fair compensation, and ensuring access to proper safety and medical facilities (Blustein et al., 2019). It includes individual aspirations relating to career development and employee performance (Huang et al., 2022). The key to retaining employees involves creating enabling work environments with fair policies and procedures (Duffy et al., 2019). To achieve employee engagement, organisations should offer jobs promoting work-life balance, safety, health, and adequate pay (Navajas-Romero et al., 2022).

The absence of decent work manifests as precarious employment, characterized by instability, unpredictability, and insecurity (McWha-Hermann et al., 2025). Employees in precarious employment experience job insecurity, which leads to financial instability and stress (Doshi et al., 2025). Traditional employment benefits, including health insurance, paid time off, and retirement plans, remain unavailable to insecure workers (Kalleberg, 2018). These workers often find themselves in unbalanced work-life situations, juggling multiple jobs or working inconsistent hours to meet basic needs.

#### 2.1.2 The social exchange theory

According to Blau’s 1964 social exchange theory, individuals form partnerships such as work relationships with the anticipation of receiving reciprocal advantages (Wang et al., 2019b). In the context of the present research, employees who overall believe that their company offers them a decent work experience characterized by key aspects such as equitable compensation, mentoring and coaching, and opportunities for professional development are more inclined to demonstrate higher levels of commitment and remain with the organisation for a longer period (Ali et al., 2024; Saurombe et al., 2017). Kaan Namal et al. (2024) highlight how the social exchange theory posits that individuals are more inclined to stick with their current employment and organisations when the prevailing norms align with their personal beliefs. Employees generally exhibit enhanced performance and heightened commitment to their work when they perceive fair treatment and recognition for their contributions. According to the social exchange theory, employees reach their highest level of performance when they receive assistance and see value from their employers (Xuecheng et al., 2022). By applying the idea to analyse employee conduct, organisations can impose specific HRM procedures and establish distinctive social exchange partnerships. Fundamentally, if workers feel that their employers provide them with a decent work experience and that their efforts are adequately rewarded, then they will be more content with their employment and less likely to leave the company (Maphanga and Olivier, 2025; Potgieter and Snyman, 2018).

### 2.2 Employee retention in the financial services sector

Employee retention represents a critical strategic imperative for financial services organisations operating in today’s talent-driven economy (Ernest and Young, 2023). Maintaining a competitive advantage requires retaining competent workers whose expertise and core capabilities prove essential for success and sustained development (Muthuku, 2020). The financial services sector, in particular, emphasises retention due to the significant direct and indirect costs associated with attrition (Nesindande et al., 2025; Ngobeni et al., 2022).

Multiple factors influence employee satisfaction and retention in the financial services sector. Bhardwaj et al. (2020) identify working hours, conditions, pay, benefits, job design, career advancement opportunities, demographic characteristics (such as age, gender, and education level), human resources department quality, supervision, and stress as key determinants. Hammond and Coetzee (2022) emphasise that perceived retention enablers include conducive work conditions, fair compensation, and continuous career development opportunities. The strong connection between compensation, benefits, and job satisfaction has a significant impact on retention decisions within the financial services industry (Sija, 2021).

Job satisfaction correlates with increased productivity, organisational loyalty, enhanced performance, and reduced turnover (Bhardwaj et al., 2020). Furthermore, satisfied employees provide superior customer service, creating positive organisational outcomes. Conversely, job dissatisfaction and unfavourable working conditions contribute significantly to attrition (Maloba and Pillay-Naidoo, 2022). Wang and Chen (2024) found that organisations promoting decent working conditions successfully decrease high employee turnover rates. Accordingly, organisations must adopt innovative strategies to retain talent through improving work conditions, promoting supervisor support, and funding employee development opportunities (Wanyama et al., 2025).

### 2.3 The nexus between decent work and retention

The relationship between decent work and employee retention represents a complex interplay of material and psychological factors. Organisations supporting work-life balance reduce human resource management expenses by hiring and retaining top performers (Sánchez et al., 2019). Work-life balance benefits individuals by enhancing quality of life, well-being, and job performance (Atitsogbe et al., 2021). When employees experience undesirable work conditions, it contributes to their intention to leave (Wang et al., 2019a).

Decent work influences employee turnover through multiple mechanisms (Chada et al., 2022) while enhancing organisations’ competitive advantage (Huang et al., 2022). Employees perceiving their work environment as conducive demonstrate higher satisfaction, better well-being, and greater engagement (Duffy et al., 2019; Işık et al., 2019). The implementation of employee-oriented human resource management strategies fosters favourable employee perspectives of work environments (Huang et al., 2022). Financial services organisations must continually revise their retention approaches to keep pace with evolving employee demands in today’s dynamic business environment.

## 3 Methodology

The following subsections of this paper outline the philosophy of the research, the technique used to develop the theory (which was deductive reasoning in this research), the various methodological decisions, the research strategy, data collection, ethical considerations, data trustworthiness and data analysis techniques.

### 3.1 Research philosophy and approach

This study employed a phenomenological paradigm to explore participants’ lived experiences related to decent work and retention. An interpretivist ontological stance enabled an understanding of participants’ subjective realities while acknowledging the constructed nature of their experiences. Epistemologically, the phenomenological paradigm helped capture the essence of participants’ experiences with decent work and retention factors, allowing deep exploration of meaning-making processes within their organisational context.

A qualitative research approach was selected as most appropriate given the study’s purpose to describe conditions and occurrences (Creswell and Poth, 2018). This approach enabled a rich and detailed exploration of participants’ perceptions and experiences of decent work, and the complex factors influencing their decisions to stay with their organisation based on these perceptions and experiences.

### 3.2 Research strategy

A case study strategy was adopted in this research, following the authors’ identification of increasing retention challenges related to decent work factors in the South African financial services sector, and particularly, within the selected financial services institution where this research was conducted. As supported by Annamalah et al. (2025) and Takahashi and Araujo (2020), case study research is often praised for its ability to enable the profound exploration and contextual understanding of intricate real-life scenario phenomena, especially in business and social science research. Case study research consequently encourages more practical and customized solutions to specific challenges, while laying a robust foundation for their further exploration in similar, and, where applicable, diverse contexts (Takahashi and Araujo, 2020). We were able to achieve this through the unique and context-specific recommendations catered to the institution explored in this research, and these recommendations can be interrogated and tested by researchers in similar or other environments, in relation to the research topic.

### 3.3 Research participants and sampling

The study population comprised 17 employees from a South African financial services organisation, selected through purposive non-probability sampling. This sampling technique enabled the identification of information-rich cases, providing relevant insights into the research phenomenon (Nayak and Singh, 2021). Participants were drawn from three departments: contact centre (5), operations (7), and support (5), ensuring diverse perspectives across organisational functions.

The sample consisted of 11 permanent and 6 temporary employees, deliberately selected to capture experiences across various employment categories. Employment duration ranged from approximately 1 year to over two decades. This variation ensured perspectives from both newcomers and veterans, providing insights into the perceptions of how decent work affects retention and how these perceptions differ based on the length of tenure within the financial services sector.

Educational qualifications varied widely from matric certificates to basic degrees, advanced degrees and professional certifications, including RE1, RE5, and the South African Institute of Chartered Accountants (SAICA) accreditation. According to the South African Qualifications Authority (SAQA) (2025), matric certificates are awarded to high school leavers upon completion, basic degrees include undergraduate qualifications, advanced degrees include graduate/postgraduate qualifications from honours and postgraduate diplomas to doctoral level, and vocational accreditations are garnered from professional bodies like the South African Institute of Chartered Accountants (SAICA). Most participants actively pursued further studies, indicating a strong orientation toward personal development. Working arrangements reflected contemporary trends, with most participants operating in hybrid models combining office and remote work, while some maintained traditional office-based positions or specialised field-based roles.

### 3.4 Data collection

Semi-structured interviews, lasting approximately 60 min on average, were conducted with each participant, allowing sufficient time for a deep exploration of their experiences while respecting their schedules. This is effective for qualitative research (Ugwu et al., 2021). Interview questions were developed in line with the research objectives, as influenced by the literature review which emphasised aspects such as fair compensation, job security, career advancement opportunities and work-life balance as fundamental to both decent work and employee retention (Demiral et al., 2022; Sija, 2021; Wang et al., 2019a). Semi-structured interviews provided flexibility to explore the developed themes while maintaining focus on research objectives, as well as to probe interesting responses, while maintaining consistency across interviews. Examples of some of the questions asked during the interviews are as follows: in your view, what are the key elements or qualities associated with “decent work”; what are the most important components of a job that would make you feel valued as an employee; based on your experiences, what are the general working conditions in the financial services sector; how do you experience fairness in terms of compensation and promotion in your current job; What factors would make you want to stay with your current employer; What mechanisms would you suggest to enhance decent work and improve employee retention in your organisation?

Most of the interviews took place in the boardrooms that were prior booked at the selected organisation’s premises to ensure privacy during the conversations. However, based on the preference and availability of some participants, some of the interviews were held virtually on Microsoft Teams. The interviews were audio-recorded with participants’ consent, firstly, by signing the informed consent forms and secondly, by confirming consent on record at the start of each interview. Each interview was transcribed verbatim to preserve the authenticity of responses. Field notes captured nonverbal cues, emotional expressions, and contextual factors that potentially influenced participants’ responses. Lastly, pseudonyms were assigned to protect participants’ identities, creating an open environment for honest expression about potentially sensitive workplace issues.

### 3.5 Ethical considerations

Following the approval of the research proposal of the study, the authors applied for ethical clearance from the University of Johannesburg’s (UJ) Department of Industrial Psychology and People Management Research Ethics Committee before commencing with the study. Prior to applying for ethical clearance, the authors submitted a letter to the HR director of the selected South African financial services sector, seeking authorisation to conduct the study there. The authors guaranteed the preservation of anonymity for both the participating organisation and the individual participants in accordance with the Protection of Personal Information (PoPI) Act of South Africa. Participation was optional, and the participants were assured of their freedom to withdraw at any point during the research process if they wished to do so. All participants received information regarding the purpose and requirements of the study prior to their involvement, and the researchers asked them to sign a consent form that would guarantee their anonymity. Participation in the study was voluntary. The researchers ensured that the potential benefits of the study outweighed any potential risks to participants, and the researchers divided the advantages and disadvantages of the study equitably among all the participants. The gathered data was utilised solely for the specific objectives of this study. The authors strove to ensure that participants would suffer no harm during the study. To preserve the participants’ integrity, the researchers stored the study’s data in a locked safe at home which will be kept for a minimum period of 5 years in line with UJ’s research data storage stipulations.

### 3.6 Data trustworthiness

The authors attempted to remain objective and avoided influencing the study’s outcomes by refraining from asking tricky or biased questions and not imposing their own beliefs on the participants during the interviews (Creswell and Poth, 2018). In addition to providing a thorough description of the research methodology and the participants’ backgrounds, the researchers conducted the study in a manner that would enhance its credibility (Stahl and King, 2020). The researchers ensured that all interview materials, including transcripts and conclusions, were stored in a secure location for future reference and auditing. The researchers aimed to sustain sufficient reflexivity throughout the study procedure (Olmos-Vega et al., 2023). The researchers listened attentively to gain a comprehensive understanding of how participants encountered and experienced the event that was studied, without forming biased opinions (Adler, 2022). The authors also provided detailed descriptions of the research methods to ensure the research process and findings can be more easily transferable to other research contexts.

### 3.7 Data analysis

Data analysis followed Braun and Clarke's (2021) six-step thematic analysis approach: familiarisation with data through multiple readings, generating initial codes systematically across the dataset, searching for themes by collating codes into potential themes, reviewing themes against coded extracts and the entire dataset, defining and naming themes to capture their essence, and producing the report with compelling extract examples. Deductive reasoning techniques were primarily used to code the data (where the literature was mostly used to underpin the development of the empirical findings). Deductive reasoning in qualitative data analysis involves a theory-driven development of the research findings, whereby existing knowledge is used to better understand the findings, as opposed to inductive reasoning techniques, which involve developing the findings from scratch in a data-driven fashion (Braun and Clarke, 2021). All authors were involved in the data analysis, with each conducting independent analyses, which were recorded from start to finish by each author in separate hardcopy journals. These analyses were ultimately collectively discussed and merged upon reaching consensus regarding the final encapsulation of the themes. The initially independently developed themes were fundamentally similar among authors, with consensus only having to be reached concerning the phrasing of each theme.

Manual analysis was deliberately chosen over software-assisted methods, enabling a profound engagement with data and allowing the authors to remain closely attuned to participants’ perspectives while collecting subtleties and contextual implications that automated methods might overlook (Korstjens and Moser, 2018). Although time-consuming, manual analysis preserved interpretive depth essential for qualitative research, ensuring genuine portrayal of participants’ experiences through direct researcher interaction with data (Busetto et al., 2020).

## 4 Findings

The findings are firstly presented in terms of the participants’ biographical data collected, and secondly in accordance with the two research objectives which were: to understand perceptions of how decent work affects employee retention and to identify factors that contribute to employee retention in the selected financial services institution. Two main themes were developed in this research. The first theme, featuring four sub themes, relates to the first research objective and the second theme, featuring six sub themes, relates to the second research objective. Each sub theme is supported by participant narratives that illuminate the complex realities of working in the financial services institution.

### 4.1 Biographical data of the research participants

With careful efforts taken not to render the research participants easily identifiable nor violate their privacy (in accordance with the PoPI Act of South Africa), we collected biographical information to enhance the richness and contextualization of the findings. Such data collected included participants’ gender, role occupied, years of experience in the financial services sector, employment type, as well as their employment model. Table 1 depicts participants’ profiles in this regard.

**Table 1:** Participants biographical data.

Pseudonym	Gender	Roles/responsibilities	Tenure in the financial services	Employment type	Employment model
P1	Female	Contact Centre—Client Services	1 year	Permanent	Hybrid
P2	Female	Contact Centre—Client Services	4 years	Permanent	Hybrid
P3	Female	Contact Centre—Client Services	5 years	Permanent	Hybrid
P4	Male	Contact Centre—Client Services	23 years	Permanent	Hybrid
P5	Female	Operations—Admin	17 years	Permanent	Hybrid
P6	Male	Client Services—Branch Admin	24 years	Permanent	Traditional, office-based
P7	Male	Support—Finance	12 years	Permanent	Hybrid
P8	Female	Operations—Broker Support	13 years	Permanent	High mobility hybrid
P9	Female	Operations—Sales	34 years	Permanent	Hybrid
P10	Male	Operations—Sales	13 years	Permanent	High mobility hybrid
P11	Female	Support—Admin	1 year 5 months	Temporary	Hybrid
P12	Male	Support—Legal	5 years	Temporary	Traditional, office-based
P13	Female	Support—HR	1 year 7 months	Temporary	Hybrid
P14	Female	Support—Admin	12 years	Temporary	Traditional, office-based
P15	Female	Support—Admin	15 years	Temporary	Traditional, office-based
P16	Female	Support—Admin	11 years	Permanent	Traditional, office-based
P17	Female	Support—Admin	8 years	Temporary	Traditional, office-based

Source: authors’ own illustration.

The research sample was generally sufficiently representative of both male (five) and female employees (12), although the authors noted an overall increased willingness to participate among females than males. This, however, is not necessarily representative of the broader male–female employee ratios in the organisation.

Most of the participants (8) were from various divisions of support staff, ranging from admin, finance, HR, and legal; while four of the participants worked in the Contact Centre-Client Services division, another four worked in various areas of the Operations division, and one worked in Client Services as a Branch Admin.

The job experience of the participants ranged from limited to extensive. The extensive experience category is associated with participants who have 16 years or more tenure. In this category, P2 and P15 each had 15 years of experience, P4 had 23 years of experience, P5 had 17 years of experience, P6 had 24 years of experience, while P9 had the longest tenure of 34 years of experience. Moderate experience is associated with participants with 6-15 years tenure. In this category, P3 had 5 years of experience, P7 and P14 each had 12 years of experience, P17 had 8 years of experience, P8 and P10 had 13 years of experience, P16 had 11 years of experience, while P2 and P15 each had 15 years of experience. The limited experience category is associated with participants with 5 or less years of tenure. In this category, P1 had 1 year experience, P11 and P13 had less than 2 years of experience, while P12 had the longest tenure of 5 years.

There are two types of employment arrangements represented in participants’ biographical information collected, namely permanent and temporary. This was done to obtain enough data around the perceptions of decent work from individuals who were not employed on a full-time contract. When this research was conducted, 11 of the participants were employed on a permanent basis, and six were employed on temporary contracts. Based on the sample, this distribution suggests the financial services’ institution’s noteworthy efforts to minimize casual and precarious work arrangements.

Based on the data, work arrangements among participants showed a continued shift toward flexible models, supporting similar findings by Chigbu and Nekhwevha (2023), Esieboma (2020) and Saurombe et al. (2022). Six employees (P6, P12, P14, P15, P16, P17) maintained traditional fully office-based positions, while the majority worked under hybrid arrangements. Nine participants (P1, P2, P3, P4, P5, P7, P9, P11, P13) follow standard hybrid schedules, splitting time between office and remote work, and two others (P8, P10) represent a specialized hybrid model that incorporates significant field work, spending considerable time “on the road” visiting clients and broker offices. This distribution indicates that hybrid work has become the predominant arrangement, with financial services organisations adapting to include both remote and field-based components.

### 4.2 Theme 1: perceptions of how decent work affects employee retention

**Figure 1:**
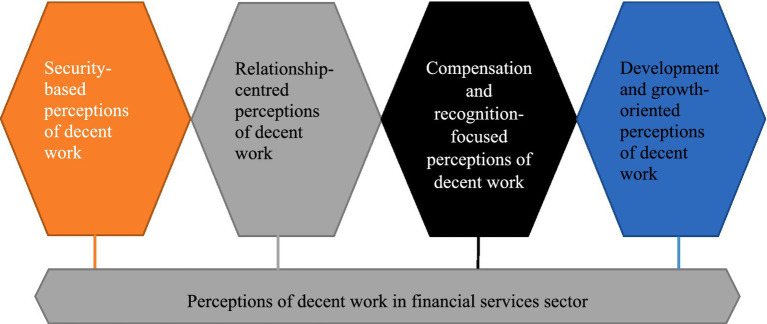
Perceptions of decent work in the financial services sector (authors’ own illustration).

Four distinct perceptions of decent work were developed from the thematic analysis, each representing different dimensions of how employees conceptualise and experience decent work within their organisational contexts (Figure 1).

#### 4.2.1 Sub theme 1: security-based perceptions of decent work

Job security was the predominant sub theme in participants’ conceptualisation of decent work, with employment stability viewed as fundamental to personal and professional well-being. This perception was particularly pronounced among temporary employees who experienced heightened vulnerability in their employment relationships.

**P7**, a permanent employee, articulated the centrality of job security:

*"Job security is critical because I still have a long way to go before retirement. So, if I were to look for a job or a promotion, I would need something stable that will keep me until I go on my retirement."*

Temporary employees expressed acute awareness of their precarious position. **P12**, who had been temporary for over 5 years, revealed:

*"I have been a temporary worker for more than 5 years; my hopes of being taken permanently are what keep me in the organisation."*

The responses in this theme indicate that job stability is a vital prerequisite for decent work for all the participants. Both permanent and temporary employees consistently recognised employment stability as being crucial; however, temporary workers articulated this necessity with greater urgency owing to their precarious employment status. In the absence of fundamental security, participants find it challenging to see their employment as being respectable, irrespective of other favourable elements in their workplace.

#### 4.2.2 Sub theme 2: relationship-centred perceptions of decent work

Participants consistently emphasised the quality of workplace relationships, particularly with management, as central to their understanding of decent work. This theme encompasses respect, support, understanding, and the overall interpersonal dynamics shaping daily work experiences.

**P4**, captured this sentiment by referencing a well-known adage:

*"People do not leave companies, they leave managers."* He emphasised the importance of clear expectations and alignment with company requirements, stating that decent work revolves around a good understanding with colleagues, particularly managers.

**P5** described decent work as a

*"happy environment"* and a *"conducive environment where company values, integrity, and teamwork are respected."* Her perspective focused on workplace atmosphere rather than specific job attributes, highlighting the importance of organisational culture in shaping decent work experiences.

#### 4.2.3 Sub theme 3: compensation and recognition-focused perceptions

Fair compensation and recognition proved to be essential components of decent work, with participants emphasising both monetary rewards and acknowledgement of contributions. This sub theme reveals how financial security and validation intertwine in employees’ conceptualisation of decent work.

**P9** perceived fair exchange for services rendered as one of the qualities of decent work, and remarked:

*“So, decent work would be I come in with my service. OK, I get paid for it. A fair wage or fair salary for what I put into it, okay. Moreover, the conditions, you know, the basic conditions of labour are there.”*

**P3** elaborated on the importance of recognition as one of the qualities of decent work, by stating:

*“Recognition, I believe, no matter how small, especially if they can see that there is improvement... Moreover, they say, ‘Wow, I am glad that you took my advice; I am glad that you have improved.’ I appreciate that, and I am seeing some growth.”*

Recognition extended beyond formal programs to include simple acknowledgements. **P10** stated:

*"Recognition is the biggest thing in any role. Recognition goes a very long way, even if it is for the smallest thing - just a pat on the back or thank you means a lot."*

#### 4.2.4 Sub theme 4: development and growth-oriented perceptions

Professional development opportunities were viewed as crucial components of decent work, with participants emphasising access to training, education, and career advancement as fundamental to their employment experience.

**P6**, combined industry-specific values with career growth aspirations:

*"Decent work involves progressing into management positions with increased responsibility while maintaining honesty and integrity in financial services."*

**P10** emphasised broader development principles:

*"Respect for workers' rights, work-life balance, equal opportunities without discrimination, and job security"* formed his perspective, incorporating both workplace justice principles and practical stability concerns.

### 4.3 Theme 2: factors contributing to employee retention

**Figure 2:**
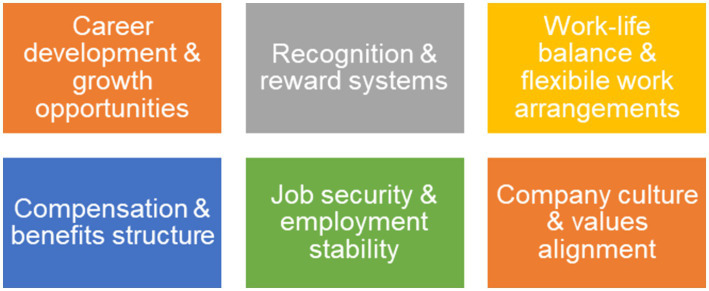
Factors contributing to employee retention (authors’ own illustration).

Six critical retention factors were developed from the analysis, each representing different mechanisms through which organisations can enhance employee commitment and reduce turnover intentions (Figure 2).

#### 4.3.1 Sub theme 1: career development and growth opportunities

Career development and growth opportunities were found to be significant retention factors, with participants consistently citing access to learning and advancement as directly influencing their decision to remain with the organisation. This sub theme encompasses formal education funding, skills development programs, and internal mobility opportunities.

**P1** described the scope of available professional development:

*"The opportunity that they give you - if you want to study, there is funding for you, and the opportunities are available in the company."*

**P2** elaborated on the range of developmental opportunities:

*"The company offers programs for learnerships, and they allow you to do your NQF level 4 and NQF level 5. The company also offers bursaries for people to apply for their studies to be funded."*

**P17**, despite temporary employment status, felt motivated to stay because of qualification advancement opportunities:

*"Yes, because once you get your qualifications, for sure you will grow within the company. That gives me hope, which is why I do not consider leaving. I need to get my regulatory qualifications so I can move up."*

#### 4.3.2 Sub theme 2: recognition and reward systems

Recognition and reward systems were revealed to be powerful retention mechanisms, with participants consistently citing acknowledgement of achievements as influencing their commitment to the organisation. This theme illustrates how recognition programs create transformative effects on workplace dynamics and employee engagement.

**P10** underlined the vital significance of recognition:

*"Recognition is the biggest thing in any role. Recognition goes a very long way, even if it is for the smallest thing - just a pat on the back or thank you means a lot."*

**P16** provided concrete evidence of recognition’s impact on retention:

*"People do not talk about leaving anymore. Since I went on the incentive trip overseas and won the annual recognition award, people became motivated to do better because they realised they could also get recognised. It changed their attitude - they are now positive."*

The cumulative effect of sustained recognition strengthened commitment. **P16** shared:

*"When I got recognised multiple times by the company, I realised they can see my effort and that I am working beyond my current job. I won the annual recognition awards three times and was the ultimate winner last year, which earned me a trip overseas."*

#### 4.3.3 Sub theme 3: work-life balance and flexible work arrangements

Work-life balance and flexible work arrangements proved to be key retention factors, with participants expressing how workplace flexibility contributed to their decision to remain with the organisation. This sub theme examined how flexible arrangements, remote work policies, and work-life integration initiatives influence organisational commitment.

**P3** outlined how remote work policies favourably impact retention:

*"Working from home is perfect for me. That 5,000 rand I would have spent on transport, I can use to pay my sister's residence fees and give her spending money. There is a level of trust here - most companies would not trust you to work from home and only come in two days, which has kept me happy."*

**P5** highlighted flexibility’s importance for family responsibilities:

*"What makes me stay is the flexible work environment, which is really helpful, especially with raising children."*

The ability to manage personal commitments while maintaining productivity proved crucial. **P2** explained:

*"The company allows us to balance our personal lives with work commitments. When you have family emergencies or need to attend to personal matters, there's understanding and flexibility."*

#### 4.3.4 Sub theme 4: compensation and benefits structure

Compensation and benefits were revealed as decisive retention factors, operating on principles of fairness, equity, and organisational commitment to employee financial well-being. The data suggests adequate compensation depends on perceived fairness and equity rather than solely competitive rates.

**P6** highlighted how organisational support during challenging times influenced retention:

*"Getting our bonus during COVID. That motivated us a lot because it showed that the company cares. It did motivate us to work harder, and yeah, and no salary cuts, that also helped us a lot to keep going and want to stay."*

Employees valued transparent salary structures, performance-based rewards, and comprehensive benefits packages. The consistency in compensation practices, even during economic challenges, demonstrated organisational commitment that strengthened employee loyalty.

#### 4.3.5 Sub theme 5: job security and employment stability

Job security and employment stability were evidenced as fundamental retention factors, creating both immediate psychological comfort and supporting long-term life planning. The findings revealed a stark divide between permanent and temporary employees’ experiences.

**P16** underscored job security’s role in facilitating future planning:

*"Job security is critical because I still have a long way to go before I get to retirement. So, if I were to look for a job or a promotion, I would need something stable that'll keep me until I go on my retirement."*

**P12** highlighted how hope for permanent employment influenced retention:

*"I have been a temporary worker for more than 5 years; my hopes of being taken permanently are what keep me in the organisation."*

**P15** echoed similar sentiments:

*"I have the hope that the organisation will eventually take us permanently, that is why I am still in the company."*

#### 4.3.6 Sub theme 6: company culture and values alignment

Company culture and values alignment proved to be influential emotional retention factors, creating deep, meaningful connections between employees and their organisation extending beyond transactional employment relationships.

**P4** described motivation through client-focused approaches during COVID-19:

*"The company allowed some employees to send personalised condolences messages to clients during the COVID period when they lost a loved one. I told myself that if people can do this and the company actually allows them to do it, why wouldn't I want to be part of something like that or a company like that?"*

**P16** captured cultural retention through emotional connection:

*"I think the company feels like home. I like the current organisation's culture - it feels like home. I like the way they serve their clients."*

**P5** described positive workplace environment impact:

*"It is like a happy environment where we respect the company's values, maintain integrity, and practice teamwork. I would call it a conducive organisational culture and environment."*

## 5 Discussion

This research aimed to understand the perceptions of how decent work affects employee retention in a South African financial services institution and to identify the factors that contribute to employee retention within the organisation. The discussion section of this paper follows the outline of the research objectives.

### 5.1 Objective 1: to understand the perceptions of how decent work affects employee retention in the financial services institution

The findings reveal a complex and multifaceted relationship between perceptions of decent work and retention factors in the financial services sector. The identification of four distinct perceptions of decent work - security-based, relationship-centred, compensation-focused, and development-oriented - demonstrates that employees conceptualise decent work through multiple lenses, each reflecting different priorities and organisational experiences.

The predominance of security-based perceptions aligns with literature highlighting job insecurity as a significant concern in casualised labour markets (Kalleberg, 2018; Kalleberg and Vallas, 2018). The stark divide between permanent and temporary employees’ experiences underscores the precarious nature of contemporary employment in the sector, where temporary workers endure years of uncertainty hoping for permanent positions. This finding corroborates Hughes et al.'s (2021) observation that precarious workers occupy disadvantaged positions with limited influence over their work environment.

The emphasis on supportive management and workplace relationships as central to decent work perceptions corroborates research suggesting that leadership quality significantly impacts employee experiences and retention decisions (Van der Merwe et al., 2020). The finding that “people do not leave companies, they leave managers” resonates throughout participants’ narratives, highlighting the crucial role of management in creating or destroying decent work conditions. This aligns with research demonstrating that supportive supervision enhances job satisfaction and reduces turnover intentions (Bhardwaj et al., 2020). These findings strongly support Dabai and Novel’s (2022) observation that informal workers in the banking sector did not experience decent working conditions compared to their permanent counterparts.

### 5.2 Objective 2: to identify factors that contribute to the retention of employees in the financial services institution

The identification of six critical retention factors provides a nuanced understanding of the mechanisms through which organisations can enhance employee commitment. Career development opportunities were found to be particularly powerful, with both formal education funding and informal skills development contributing to retention. This finding supports Hammond and Coetzee's (2022) emphasis on continuous career development as a retention enabler. However, the systematic exclusion of temporary workers from development opportunities creates a two-tiered system, contradicting decent work principles of equality.

Recognition and reward systems demonstrated transformative effects on workplace culture and retention. The finding that recognition “changed attitudes” and stopped people “talking about leaving” illustrates how acknowledgement programs create ripple effects throughout organisations. This supports the literature emphasising the role of recognition in enhancing employee engagement and organisational commitment (Xuecheng et al., 2022). The effectiveness of both monetary and non-monetary recognition suggests organisations should develop multi-layered acknowledgement systems addressing diverse employee needs.

Work-life balance and flexible arrangements have proven to be increasingly critical retention factors, particularly in the post-pandemic era. Participants’ appreciation for remote work opportunities and the flexibility they offer in managing personal responsibilities reflects global trends toward hybrid working models. The financial benefits of remote work, such as reduced transport costs, add an economic dimension to the retention impact of flexibility. This finding aligns with research demonstrating that organisations supporting work-life balance successfully reduce turnover while enhancing employee well-being (Sánchez et al., 2019).

Compensation proved not to be mere financial consideration, but also as a recognition of value and an organisational commitment to employee well-being. For example, participants’ perceived importance of maintaining bonuses during COVID-19 demonstrated how compensation decisions during crises significantly impact employee loyalty. This finding supports Chada et al.'s (2022) assertion that decent work encompasses both material and psychological dimensions of employment. The emphasis on fairness and transparency over absolute compensation levels suggests that organisations should prioritise equitable and transparent reward systems, thus corroborating Chitsaz-Isfahani and Boustani (2014) and Mthintso et al.’s (2024) research results.

The present study’s revelation of job security as a fundamental retention factor, particularly for temporary employees, highlights the psychological toll of precarious employment. The finding that temporary workers remain with organisations for years, hoping for permanent positions, reveals both their vulnerability and organisations’ potential exploitation of this hope. This aligns with the literature on the negative impacts of precarious employment on worker well-being and life planning capabilities (Doshi et al., 2025).

Company culture and values alignment within the financial services institution created emotional connections, transcending transactional employment relationships. Participants who describe their organisation as “home” and express pride in corporate social responsibility initiatives demonstrate the culture’s powerful retention influence. This finding supports research emphasising the role of organisational culture in creating a sense of belonging and commitment. The diversity of cultural elements contributing to retention - from client service orientation to transparency in communication - suggests that organisations should cultivate multifaceted cultures that address various employee values.

The interplay between perceptions of decent work and retention factors reveals important theoretical and practical implications. Security-based perceptions of decent work are directly linked to job security as a retention factor, while relationship-centred perceptions connect to the importance of management quality and organisational culture (Gandi and Saurombe, 2025; Maloba and Pillay-Naidoo, 2022). Similarly, development-oriented perceptions align with career advancement opportunities as retention mechanisms (Gandi and Saurombe, 2025). This interconnection suggests that organisations seeking to enhance retention must address decent work comprehensively rather than focusing on isolated factors.

## 6 Practical implications

The findings provide actionable insights for financial services organisations seeking to enhance retention through the principles of decent work. The authors posit the following recommendations based on the research findings:

### 6.1 Abolish the two-tier employment system

The financial services sector must restructure their employment practices fundamentally to eliminate the permanent-temporary employees divide that undermines decent work. This can be achieved by establishing clear pathways and timeframes to transition temporary workers to permanent employment after a maximum of 12 months. Organisations should extend basic benefits such as medical aid, pension contributions, and overtime pay to all employees, regardless of their contract type or employment status. This structural reform is foundational, as other interventions cannot succeed while systemic inequality persists. Moreover, regulatory bodies should enforce limits on the use of temporary contracts for inherently permanent roles, thereby preventing the exploitation of workers through perpetual temporary employment.

### 6.2 Extension of access to developmental opportunities

All employees, regardless of employment status, should have equal access to training and development programs. Organisations should provide study assistance and bursary schemes to long-term temporary workers, recognising that investing in their development benefits both parties. This includes implementing comprehensive AI and digital skills training programs to address technological disruption in the sector. Development programs should focus on portable, formal qualifications that enhance both organisational capability and individual career security. Additionally, mentorship and career guidance should be readily available to all employees to support their professional development and growth.

### 6.3 Developing comprehensive recognition systems

Evidently, opacity in compensation undermines trust and the principles of decent work. The financial services sector should establish transparent salary scales and progression criteria, ensuring that employees understand how their compensation compares to market rates and internal benchmarks. Moreover, position-based rather than individual-based pay structures should be implemented to ensure the principle of equal pay for equal work. Financial services sector organisations should consider implementing recognition programs that acknowledge both significant achievements and everyday contributions, with particular attention being paid to ensuring that temporary workers are included in recognition initiatives. Lastly, regular salary reviews and adjustments should be conducted to maintain competitive and fair compensation.

### 6.4 Institutionalizing flexible work arrangements

Flexible work has proven to be beneficial for both productivity and employee well-being. Organisations should formalise hybrid working policies that apply equitably to all employees, and not merely senior or permanent staff. This includes providing necessary technology and support for practical remote work, recognising that flexibility enhances work-life balance and reduces commuting costs, which is significant in the South African context. However, implementation must consider the digital divide and infrastructure challenges such as load shedding and unstable internet connectivity, ensuring that flexible work arrangements do not inadvertently exclude or disadvantage certain employee groups. Financial services sector organisations should implement clear guidelines for flexible work eligibility, and expectations should be established and communicated, offering employees a choice rather than mandates, while recognising that different employees thrive in different work environments.

### 6.5 Foster genuine inclusive organisational cultures

Cultural transformation must be backed by structural equity to be meaningful. Organisations need to ensure that ‘family’ rhetoric is matched by inclusive practices that treat all employees with equal dignity and respect. This requires training managers in inclusive leadership practices that recognise and value all employees regardless of contract type. In addition, psychological safety must be created, where employees can voice concerns without fearing that their contract will not be renewed. Regular culture audits should be conducted to assess whether the adopted values align with the lived experiences of employees across all categories. Finally, employee feedback mechanisms should include temporary workers in decision-making processes that affect their work.

### 6.6 Strengthen job security and employment stability

This research found job security to be the foundation upon which other elements of decent work are built. The financial services sector should provide greater employment security through longer-term contracts and transparent renewal processes. During economic downturns, organisations should prioritise retaining employees rather than defaulting to retrenchments, recognising that employment security enhances loyalty and productivity. For roles that are genuinely temporary or project-based, clear communication about contract duration and potential for extension should be provided from the outset. The financial services sector should implement succession planning and create internal promotion opportunities that prioritise existing temporary workers for permanent positions.

## 7 Limitations and recommendations

This study’s limitations provide opportunities for future research. The single-organisation focus, while enabling deep exploration of experiences within one context, limits generalizability across the financial services sector. Nonetheless, the research remains transferable to other research contexts, which is a strength associated with qualitative case study research. Future research should consider examining multiple organisations across banking, insurance, and investment sectors to identify sector-specific variations in decent work perceptions and retention factors.

The qualitative approach, while providing rich insights into lived experiences, prevents quantification of relationships between variables. Quantitative research could test the relationships identified between decent work dimensions and retention outcomes, potentially developing predictive models for turnover based on decent work indicators. Mixed-methods approaches could combine depth of understanding with statistical validation.

The cross-sectional design captures perceptions at one point in time, missing how these evolve. Longitudinal studies could examine how decent work perceptions and retention factors change over employees’ careers, potentially identifying critical junctures where interventions prove most effective. Such research could also track whether improvements in decent work conditions translate into enhanced retention over time.

The study focused on individual perceptions without examining organisational policies and practices shaping these experiences. Future research could adopt multi-level approaches examining how organisational strategies, departmental cultures, and individual characteristics interact in shaping decent work experiences and retention decisions. Comparative studies between high and low turnover organisations could identify best practices for creating decent work conditions.

The South African context, while providing important insights for emerging economies, may not reflect experiences in developed nations with different labour market conditions. International comparative research could identify universal versus context-specific aspects of decent work and retention, informing global financial services organisations operating across multiple countries.

## 8 Conclusion

This research provides valuable insights into the complex relationship between decent work perceptions and employee retention in the South African financial services sector. The identification of four distinct perceptions of decent work - security-based, relationship-centred, compensation-focused, and development-oriented - demonstrates that employees conceptualise decent work through multiple, interconnected dimensions. Similarly, the development of six critical retention-related themes from the research data including career development and growth opportunities, recognition and reward systems, work-life balance and flexible work arrangements, compensation and benefits structure, job security and employment stability, as well as alignment of company culture and values, reveals the multifaceted nature of organisational commitment factors in contemporary workplaces.

The findings highlight significant disparities between permanent and temporary employees’ experiences, revealing how employment status fundamentally shapes access to decent work conditions and retention mechanisms. Temporary workers enduring extended periods of precarious employment as they hope for permanent positions while being excluded from development opportunities and benefits, represents a critical challenge for organisations claiming commitment to decent work principles.

The interconnection between decent work perceptions and retention factors suggests organisations cannot address retention challenges through isolated interventions. Instead, comprehensive approaches addressing job security, management quality, recognition, flexibility, development opportunities, compensation, and organisational culture prove necessary. The transformative effects of recognition programs and flexible work arrangements demonstrate that even relatively modest investments in decent work conditions can yield significant retention benefits.

As the financial services sector continues evolving amid technological disruption, changing workforce expectations, and economic uncertainties, creating decent work conditions becomes increasingly critical for organisational sustainability. Organisations that successfully integrate decent work principles into their employment practices will likely enjoy competitive advantages through enhanced retention, improved employee engagement, and stronger organisational cultures.

The research contributes to theoretical understanding by demonstrating how decent work theory applies differentially across employment categories, highlighting the importance of inclusive approaches to creating equitable work conditions. Practically, it provides evidence-based strategies for enhancing retention through comprehensive decent work interventions addressing diverse employee needs and expectations.

Ultimately, this research demonstrates that employee retention in the financial services sector requires more than competitive compensation or isolated perks. It demands fundamental commitment to creating work environments where all employees, regardless of employment status, experience security, respect, fair rewards, and opportunities for growth. By embracing decent work principles comprehensively, organisations can create sustainable employment relationships benefiting both employees and organisational performance in an increasingly challenging labour market.

## Data Availability

The raw data supporting the conclusions of this article will be made available by the authors, without undue reservation.
